# Exploring Unique Extracellular Vesicles Associated Signatures: Prognostic Insights, Immune Microenvironment Dynamics, and Therapeutic Responses in Pancreatic Adenocarcinoma

**DOI:** 10.1155/2024/2825971

**Published:** 2024-08-24

**Authors:** Kai Nan, Ming Zhang, Zilong Geng, Yuankai Zhang, Lin Liu, Zhi Yang, Peng Xu

**Affiliations:** ^1^ Department of Joint Surgery HongHui Hospital Xi'an Jiaotong University, Xi'an 710054, Shaanxi, China; ^2^ Department of General Practice Honghui Hospital Xi'an Jiao Tong University, Xi'an 710054, Shaanxi, China; ^3^ Department of Orthopaedics The Second Affiliated Hospital of Xi'an Jiaotong University, Xi'an 710054, Shaanxi, China

## Abstract

Extracellular vesicles play an important role in the progression of pancreatic adenocarcinoma (PAAD) through the transfer of proteins, mRNAs, and long noncoding RNAs (lncRNAs). However, the intricate interplay between extracellular vesicles-related lncRNAs and the tumor microenvironment (TME) remains poorly elucidated. Consequently, our investigation aimed to delineate the association between extracellular vesicles-related lncRNAs and the PAAD microenvironment. Initially, we identified differentially expressed lncRNAs (DELs) from The Cancer Genome Atlas (TCGA) and Genotype-Tissue Expression (GTEx) project datasets. Subsequently, we validated the expression of these DELs within extracellular vesicles and assessed their prognostic implications in PAAD using the GSE133684 and TCGA datasets. Multiomics data were analyzed comprehensively, including genomic landscape, functional annotation, immune profiles, and therapeutic responses. Differential expression of selected lncRNAs in both cellular and exosomal fractions of PAAD was further confirmed through quantitative polymerase chain reaction (qPCR). Eight DELs were identified from TCGA and GTEx datasets, and two exosomal lncRNAs exhibited a significant correlation with overall survival, warranting further investigation. Specifically, elevated expression of LINC00996 correlated positively with immune infiltration and enhanced response to immunotherapy. Conversely, heightened expression of TRHED-AS1 was associated with compromised immune cell infiltration and diminished responsiveness to immunotherapy. Our study establishes a compelling link between two extracellular vesicles-related gene signatures, prognosis, and immune infiltration in PAAD. Notably, these signatures serve as robust prognostic indicators for PAAD patients, offering valuable insights for the strategic selection of immunotherapeutic interventions.

## 1. Introduction

Worldwide, pancreatic adenocarcinoma (PAAD) ranks fourth in terms of cancer-related deaths, with 5-year survival rates not exceeding 13% [1]. Despite advancements in pancreatic cancer therapy, the postoperative outlook remains less than optimal, chiefly due to the subtle onset and elevated rates of recurrence and metastasis [2, 3]. Notwithstanding notable therapeutic advances, the prognosis for PAAD patients remains disheartening. Consequently, the identification of prognostic biomarkers assumes critical importance for predicting survival and refining treatment strategies.

Noncancerous cells, their constituents within the tumor, and molecules produced or secreted by these cells form the tumor microenvironment (TME). Dynamic interactions between tumor cells and their microenvironment affect tumor initiation, progression, metastasis, and response to treatment [4]. Extracellular vesicles, derived from endosomes, typically ranging in size from 40 to 160 nm with an average diameter of 100 nm, emerge as pivotal mediators of intercellular communication in tumors when released by both tumor and TME cells [5, 6]. The involvement of extracellular vesicles in PAAD has been extensively studied, including initiation, growth, metastasis, angiogenesis, epithelial–mesenchymal transition, immunity, and drug resistance [7, 8, 9, 10].

LncRNAs, characterized by their length exceeding 200 nucleotides and restricted protein-coding capacity, have a significant impact on the development and advancement of cancers, including PAAD [11, 12]. There is growing evidence that exosome-associated lncRNAs may play a role in the diagnosis and development of PAAD [13, 14]. Nevertheless, the exact function, prediction, and plausible molecular pathways linked to exosome-associated lncRNAs remain enigmatic within the scope of PAAD.

This study aims to elucidate lncRNA signatures in PAAD through differential expression analysis based on TCGA and GTEx databases. Subsequently, modules of extracellular vesicles-related lncRNAs were discerned using GEO datasets, and prognostic lncRNAs were extracted for further scrutiny. To uncover the molecular features underpinning the prognostic signature, a comprehensive bioinformatic analysis was conducted, incorporating multiomics data. Furthermore, drug resistance and therapeutic response were meticulously examined. Ultimately, the variance in expression of chosen lncRNAs was authenticated in both cellular models and exosomal fractions employing quantitative real-time polymerase chain reaction (qPCR).

## 2. Materials and Methods

### 2.1. Data Sources and Preprocessing

The RNA-seq transcriptomic information of 178 individuals diagnosed with PAAD, sourced from TCGA, was augmented by data from 171 healthy individuals acquired from UCSC Xena (http://xena.ucsc.edu/). To maintain consistency with PAAD data, the GTEx data were transformed from log2 (*X* + 0.001) to log2 (*X* + 1). Patients lacking survival data were excluded from the study to alleviate statistical biases. Following this, long RNA sequencing data from blood extracellular vesicles of both PAAD patients and healthy individuals were obtained from the GSE133684 dataset.

### 2.2. Identification of Prognosis-Associated DELs

To investigate the expression disparity between PAAD and normal tissues, the Wilcoxon test was used using the “limma” package in R (criteria: log2 fold change > 1, false discovery rate > 0.05). Subsequently, a heatmap was then used to visualize eight DELs. Following this, the expression data of these DELs were integrated with survival data from 176 patients and subjected to univariate Cox analysis, with a significance threshold set at *P* < 0.01. Finally, two extracellular vesicles-derived DELs associated with prognosis were discerned.

### 2.3. Gene Set Enrichment Analysis (GSEA)

The GSEA software (version 4.2.1) was used to discern predefined biological pathways that significantly differentiate high and low expression groups (*P*  < 0.01 and FDR < 0.1). Based on the Molecular Signatures Database file c2.cp.kegg.v7.1.symbols.gmt, the results were visualized using ggplot2, grid, and gridExtra R packages.

### 2.4. Investigation into Immune Infiltration and Immune Checkpoints

Validation was performed utilizing the biomarker exploration of solid tumors (BEST) portal. BEST was employed to analyze the association between prognosis, response to immunotherapy, potential drug candidates, and expression of lncRNAs in PAAD. A Spearman correlation analysis was performed between the IC50 scores and the expression of LINC00996 and TRHDE-AS1. Different sample expressions are represented on the *x*-axis, while IC50 scores are represented on the *y*-axis. The density curve on the right illustrates the distribution trend of IC50 scores, with the upper-density curve indicating the trend in gene expression distribution. The value at the top represents the correlation *P*-value, correlation coefficient, and the method of correlation calculation.

### 2.5. Isolation and Validation of Extracellular Vesicles

Extracellular vesicles were isolated from the cell culture medium through ultracentrifugation. PAAD cells, cultivated in 15-cm diameter dishes until reaching 70% confluency, underwent a series of centrifugation steps, culminating in ultracentrifugation at 110,000× *g* for 70 min at 4°C using a Beckman Coulter Type 70ti rotor. The extracellular vesicle pellets were resuspended in PBS and underwent further ultracentrifugation at 110,000× *g* for 70 min. The resulting pellet was then resuspended in 100 *μ*l PBS and stored at −80°C. For transmission electron microscopy (TEM) analysis, extracellular vesicles suspended in PBS were deposited onto formvar carbon-coated grids and stained with 2% phosphotungstic acid. The grid was air-dried and images were captured using a TEM device at 80 kV. Additionally, the size of extracellular vesicles was assessed using the NanoSight NS300 device. Proteins from lysed extracellular vesicles were subjected to immunoblotting using anti-TSG101 (#612 696, BD Biosciences) and anti-CD63 (#25682-1-AP, Proteintech).

### 2.6. RT-qPCR

The mRNA expression matrix of cell lines was acquired from the CCLE dataset (https://portals.broadinstitute.org/ccle). The *x*-axis represents different cell lines, while the *y*-axis represents the distribution of mRNA expression. For cell experiments, normal human pancreatic ductal epithelial cells hTERT-HPNE and two pancreatic cancer cells, PANC-1 and BxPC-3, were obtained from the Cell Bank of Type Culture Collection of the Chinese Academy of Sciences. HPNE and PANC-1 were cultured in DMEM with 10% fetal bovine serum (FBS) and 1% penicillin/streptomycin. BxPC-3 was cultured in RPMI medium with 10% FBS. Total RNA was extracted using the Fastgen 200 RNA isolation kit and reverse transcribed into cDNA using the Prime Script RT reagent kit. RT-qPCR was performed using the CFX Manager 2.1. The primer sequences were illustrated in Table S1.

## 3. Results

### 3.1. Identification of Prognosis-Associated Extracellular Vesicles DELs

**Figure 1:**
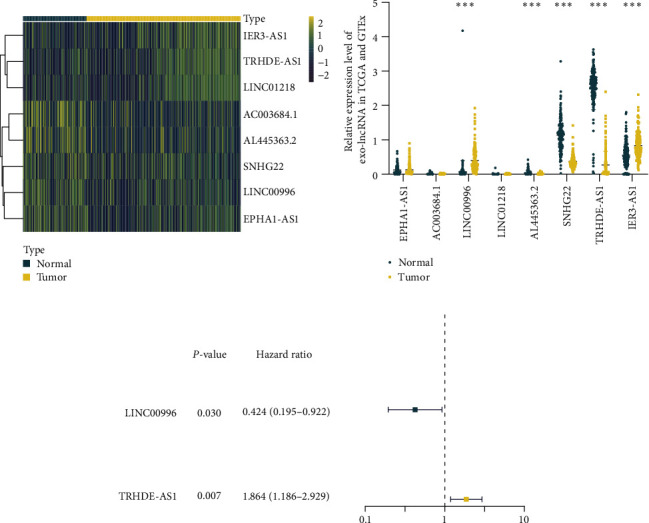
Identification of prognosis-associated exosome-derived DELs. (a) A heatmap depicting the expression of DELs based on TCGA and GTEx datasets. (b) Validation of the expression of eight DELs in extracellular vesicles using data from GSE133684. (c) A forest plot presenting prognostic lncRNAs identified through univariate Cox regression analysis.  ^*∗∗∗*^*P* < 0.001.

In total, 178 tumor samples and 171 normal samples (including 167 samples from GTEx) were obtained from UCSC Xena, based on TCGA and GTEx datasets. Subsequently, eight DELs were identified between tumor and normal samples, with five downregulated and three upregulated (criteria: log2 FC > 1, FDR < 0.05, Figure 1(a)). The expression of these eight DELs in extracellular vesicles was then validated using data from GSE133684, which consisted of 117 healthy patients and 284 PAAD patients (Figure 1(b)). Additionally, a forest plot (Figure 1(c)) depicts the results of univariate Cox analysis, illustrating the association between the expression of the identified DELs and survival outcomes in TCGA.

### 3.2. Exploration of the Correlation between LINC00996 Expression and Prognosis, Immune Correlation, and Drug Sensitivity in PAAD

**Figure 2:**
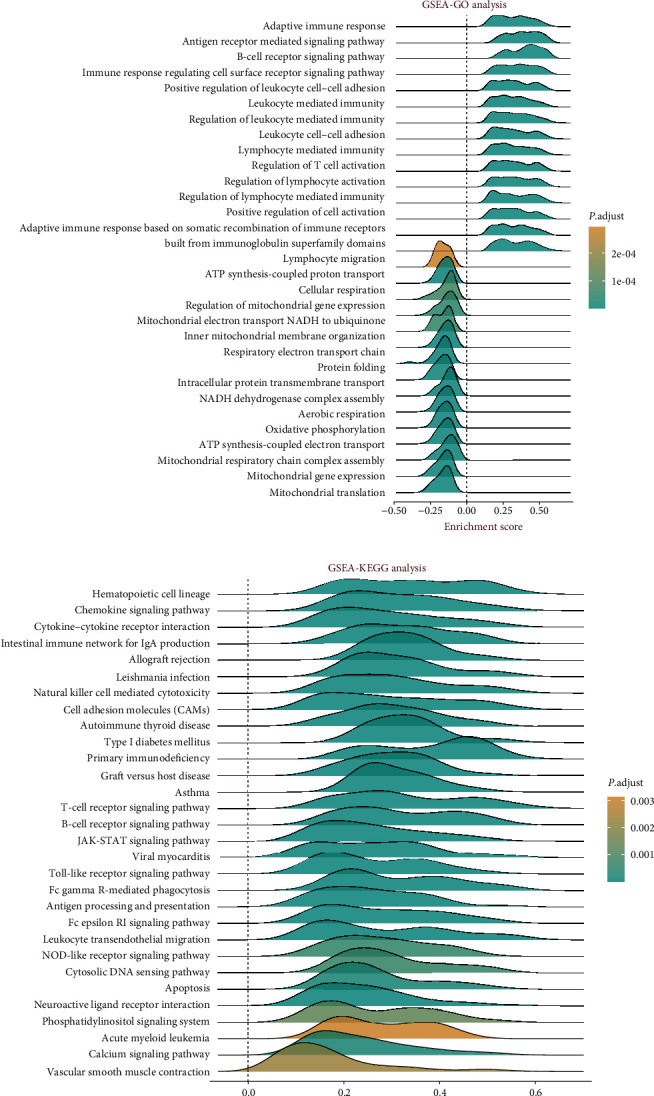
Investigation of the correlation between LINC00996 expression and prognosis, as well as immune correlation in PAAD. (a) A forest plot depicting LINC00996 expression in diverse datasets through univariate Cox regression analysis. (b) The expression of LINC00996 between diabetes and nondiabetes groups in GSE79668. (c) The expression of LINC00996 between male and female patients in GSE79668. (d) GSEA-GO analysis of LINC00996. (e) GSEA-KEGG analysis of LINC00996. (f) The correlations between LINC00996 expression and immune scores in GSE57495, GSE79668, TCGA_PAAD, and ICGC_PAAD-AU datasets.  ^*∗*^*P* < 0.05 and  ^*∗∗*^*P* < 0.01.

Initially, univariate Cox regression was conducted to explore the relationship between LINC00996 expression and prognosis across various datasets (Figure 2(a)). The analysis revealed that the high expression group of LINC00996 clustered predominantly on the left side of the null line, indicating that LINC00996 may serve as a protective factor. Subsequently, GSE79668 was utilized to examine the correlation between LINC00996 expression and clinicopathological characteristics in PAAD. Notably, significantly lower expression of LINC00996 was observed in nondiabetic and male patients in GSE79668 (Figures 2(b) and 2(c)). Furthermore, GSEA was employed based on LINC00996 expression, with a significance threshold set at a *P*-value < 0.05. As depicted in Figure 2(d), high expression of LINC00996 was predominantly enriched in pathways related to adaptive immune response, antigen receptor-mediated signaling, B cell receptor signaling, immune response regulation via cell surface receptor signaling, and positive regulation of leukocyte cell adhesion. Conversely, low expression of LINC00996 was mainly enriched in pathways related to ATP synthesis-coupled proton transport, cellular respiration, regulation of mitochondrial gene expression, mitochondrial electron transport NADH ubiquinone, and inner mitochondrial membrane organization. Additionally, high expression of LINC00996 was found to be enriched in pathways associated with hematopoietic cell lineage, chemokine signaling, cytokine receptor interaction, intestinal immune network for IgA production, and allograft rejection (Figure 2(e)). Finally, a significant correlation was observed between LINC00996 expression and immune scores across multiple datasets including GSE57495, GSE79668, TCGA, and ICGC-AU (Figure 2(f)).

### 3.3. Examination of the Correlation between TRHDE-AS1 Expression and Prognosis, Immune Correlation, and Drug Sensitivity in PAAD

**Figure 3:**
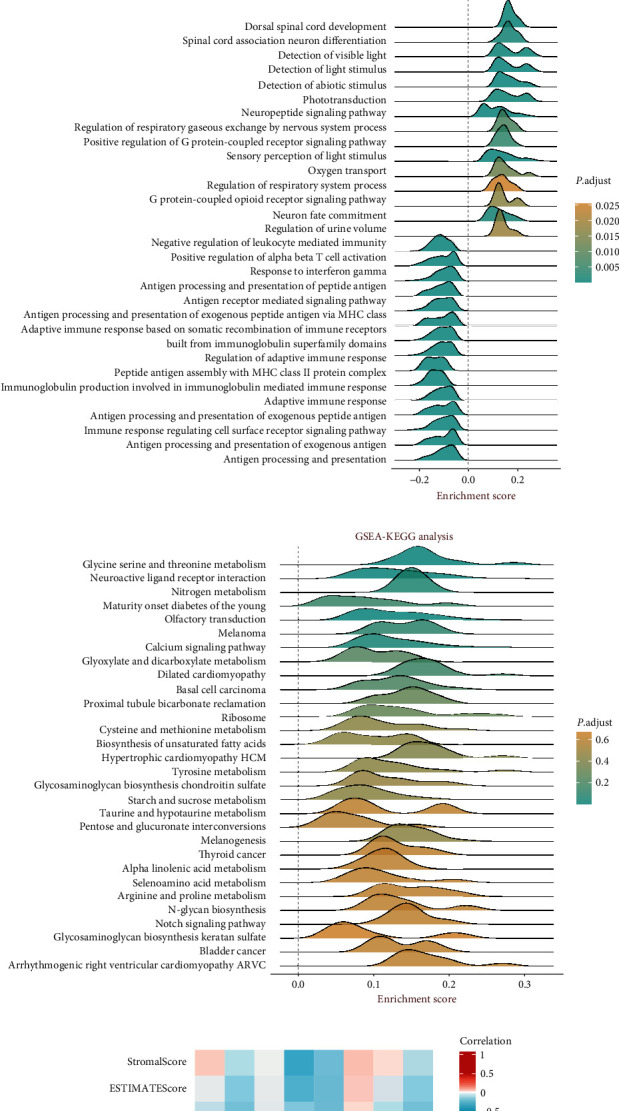
Investigation into the correlation between TRHDE-AS1 expression and prognosis, as well as immune correlation in PAAD. (a) TRHDE-AS1 expression in GSE62452. (b) A forest plot depicting TRHDE-AS1 across diverse datasets using univariate Cox regression analysis. (c) GSEA focusing on GO pathways associated with TRHDE-AS1. (d) GSEA focusing on KEGG pathways linked to TRHDE-AS1. (e) Examination of the correlations between ImmuneScore, ESTIMATEScore, StromalScore, and TRHDE-AS1 expression. (f) Investigation into the correlation between TRHDE-AS1 expression and PDCD1 expression.  ^*∗*^*P* < 0.05 and  ^*∗∗*^*P* < 0.01.

The expression data of TRHDE-AS1 were extracted from GSE62452. Subsequently, it was observed that TRHDE-AS1 expression was notably decreased in PAAD compared to healthy subjects (Figure 3(a)), consistent with the findings in extracellular vesicles depicted in Figure 1(b). Following this, univariate Cox regression analysis was conducted to examine the association between TRHDE-AS1 expression and prognosis across various datasets (Figure 3(b)). In this investigation, higher TRHDE-AS1 expression predominantly clustered on the right side of the null line in most datasets, indicating its role as a risk factor. GO analysis and KEGG pathway enrichment analysis were performed based on TRHDE-AS1 expression, employing a significance threshold of *P*-value < 0.05. The GO pathway, depicted in Figure 3(c), exhibited enrichment primarily in processes such as dorsal spinal cord development, spinal cord association neuron differentiation, detection of visible light, detection of light stimulus, and detection of abiotic stimulus. As illustrated in Figure 3(d), KEGG pathways were predominantly enriched in glycine serine and threonine metabolism, neuroactive ligand–receptor interaction, nitrogen metabolism, maturity-onset diabetes of the young, and olfactory transduction. Subsequent exploration focused on the correlation between TRHDE-AS1 expression and stromal score, ESTIMATE score, and immune score based on TCGA data.  Figure 3(e) demonstrates a negative correlation between TRHDE-AS1 expression and stromal score, ESTIMATE score, and immune score, suggesting a potential role of TRHDE-AS1 as an immunosuppressive gene. Evaluation of immune checkpoints aids in assessing the patient's immune response to immunotherapy [15]. Investigation into the correlation between TRHDE-AS1 expression and PDCD1 expression was conducted across diverse datasets including GSE62452, GSE78229, and GSE28732. As depicted in Figure 3(f), a significant correlation between TRHDE-AS1 expression and PDCD1 expression was observed.

### 3.4. Mutation Profile and Immunotherapy Response Based on LINC00996 and TRHDE-AS1 Expression

**Figure 4:**
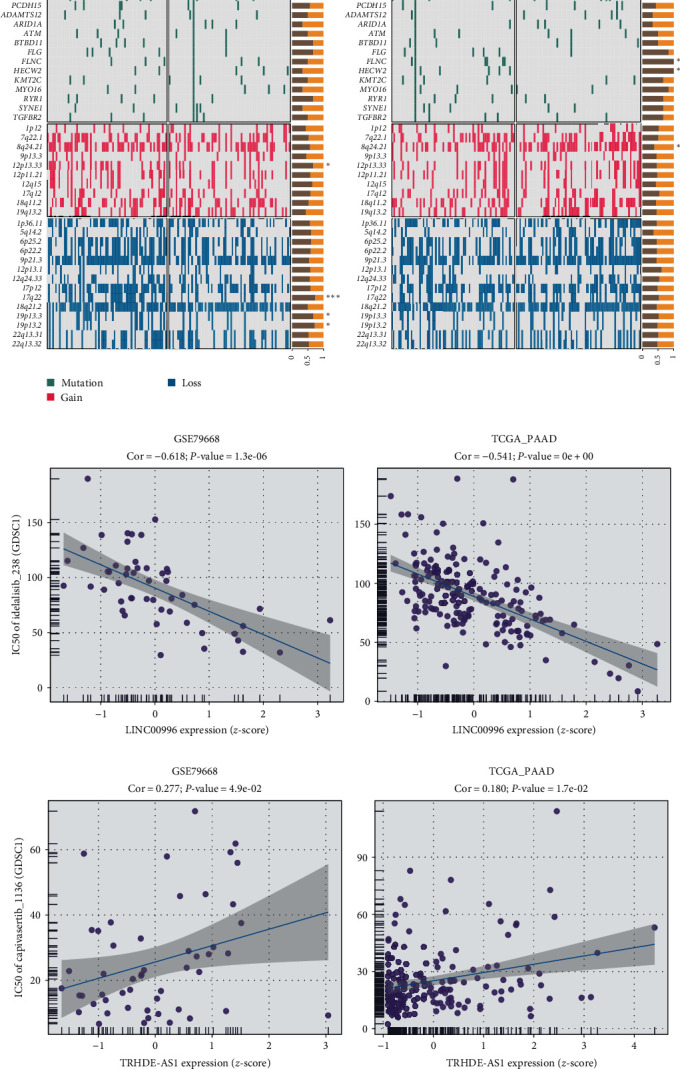
Mutation profile and drug sensitivity based on LINC00996 and TRHDE-AS1 expression. (a) Somatic landscape of different expressions of LINC00996 and TRHDE-AS1 in PAAD. (b) Correlation between IC50 of idelalisib_238 and LINC00996 expression. (c) Correlation between IC50 of capivasertib_1136 and TRHDE-AS1 expression.  ^*∗*^*P* < 0.05,  ^*∗∗*^*P* < 0.01, and  ^*∗∗∗*^*P* < 0.001.

**Figure 5:**
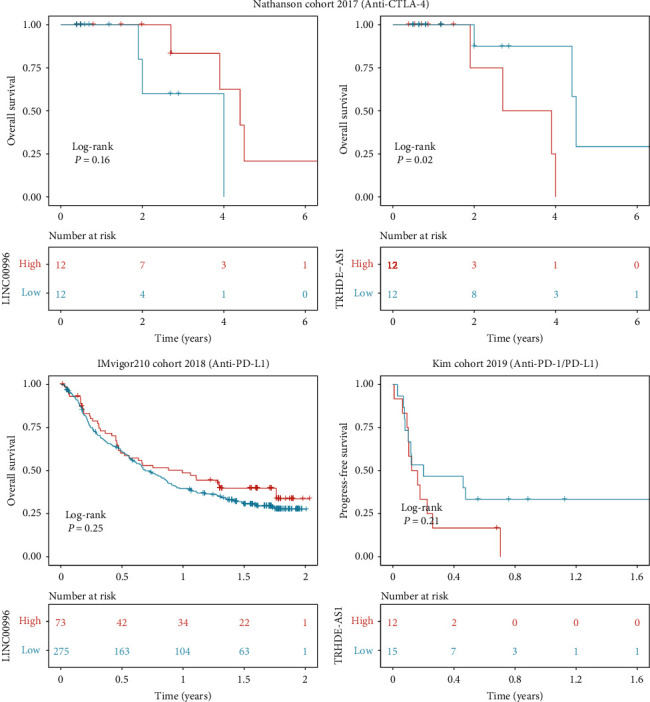
Kaplan–Meier survival curves comparing patients with varying expressions of LINC00996 and TRHDE-AS1 in the anti-CTLA4 and anti-PD-L1 cohorts.

Tumor mutational burden (TMB) constitutes a pivotal factor in tumor onset and progression, thus offering predictive value regarding the efficacy of immune checkpoint inhibitors (ICIs) [16]. Within this study, cancer-related gene mutation data were utilized to evaluate the correlation between TMB levels and the expression of LINC00996 and TRHDE-AS1. Findings demonstrated a significant rise in KRAS mutation frequencies within the high LINC00996 expression group compared to the low-expression group. Additionally, the low expression group of TRHDE-AS1 exhibited elevated FLNC and HECW2 mutation frequencies (Figure 4(a)). Considering that IC50 values reflect cellular sensitivity to chemotherapeutic agents, two chemical drugs, idelalisib_238 and capivasertib_1136, were assessed. As depicted in Figure 4(b), LINC00996 expression exhibited a negative correlation with the IC50 of idelalisib_238 in GSE79668 and TCGA datasets, suggesting the potential sensitivity of high LINC00996 expression patients to idelalisib_238. As depicted in Figure 4(c), TRHDE-AS1 expression exhibited a positive correlation with the IC50 of Capivasertib_1136 in GSE79668 and TCGA datasets, suggesting potential resistance of high TRHDE-AS1 expression patients to Capivasertib_1136. Focusing on the distribution of immune checkpoint expression, attention was directed toward anti-CTLA4 and CD274 (PD-L1) therapy in the Nathanson cohort 2014 (anti-CTLA-4), IMvigor210 cohort 2018 (anti-PD-L1), and Kim cohort 2019 (anti-PD-1/PD-L1). Results revealed that the high LINC0096 expression group exhibited prolonged survival compared to the low expression group in the anti-CTLA4 and anti-PD-L1 groups (Figure 5). Conversely, the high TRHDE-AS1 expression group demonstrated a poorer prognosis in the anti-CTLA4 and anti-PD-L1 groups.

### 3.5. Validation of the Expression and Prognostic Significance of LINC00996 and TRHDE-AS1 in PAAD

**Figure 6:**
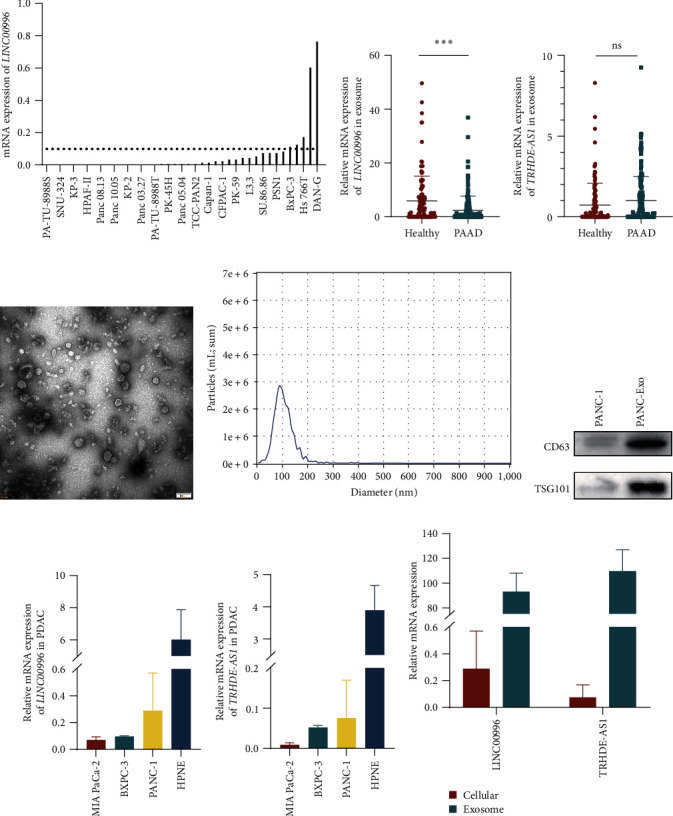
Validation of the expression and prognostic value of LINC00996 and TRHDE-AS1 in PAAD. (a) Assessment of LINC00996 mRNA expression in the CCLE database. Examination of LINC00996 (b) and TRHDE-AS1 (c) expression in extracellular vesicles from the ExoRbase database. (d) Transmission electron microscopy images of extracellular vesicles derived from PANC-1 cells, with a scale bar indicating 100 nm. (e) Nanoparticle tracking analysis depicting size distributions and the number of extracellular vesicles derived from PANC-1 cells. (f) Western blot analysis of exosome markers CD63 and TSG101 in PANC-1 cells and extracellular vesicles. Evaluation of LINC00996 (g) and TRHDE-AS1 (h) expression in PAAD cells. (i) Assessment of LINC00996 and TRHDE-AS1 expression in PANC-1 cells and extracellular vesicles.  ^*∗∗∗*^*P* < 0.001.

Initially, LINC00996 expression was examined in the Cancer Cell Line Encyclopedia (CCLE). The analysis revealed elevated expression of LINC00996 in DAN-G, Hs 766T, and BXPC-3 PAAD cells compared to others (Figure 6(a)). The ExoRbase dataset serves as a repository for extracellular vesicle (EV) messenger RNA (mRNA), lncRNA, and circular RNA (circRNA) derived from human biofluids [17]. Subsequently, the expression levels of LINC00996 and TRHDE-AS1 were validated using the ExoRbase dataset. It was observed that LINC00996 exhibited reduced expression in PAAD extracellular vesicles compared to healthy controls (Figure 6(b)). Conversely, there was no statistically significant difference in TRHDE-AS1 expression in extracellular vesicles between PAAD patients and healthy controls (Figure 6(c)). Further validation of LINC00996 and TRHDE-AS1 expression in extracellular vesicles involved the extraction of extracellular vesicles from PANC-1 cells via ultracentrifugation. The extracellular vesicles exhibited a cup-shaped morphology, ranging from 50 to 150 nm in diameter, and expressed CD63 and TSG101 markers (Figures 6(d)and 6(e)). Notably, the expression levels of LINC00996 and TRHDE-AS1 were markedly higher in HPNE cells compared to PAAD cells (Figures 6(g) and 6(h)). Subsequent investigation into LINC00996 and TRHDE-AS1 expression in extracellular vesicles derived from PANC-1 cells revealed a significant overexpression compared to PANC-1 cells (Figure 6(i)).

## 4. Discussion

Pancreatic cancer is an exceptionally malignant tumor within the digestive system. Owing to its subtle symptoms and swift disease progression, the majority of patients are diagnosed with locally advanced or metastatic disease, leading to suboptimal treatment outcomes [18]. Accumulating evidence indicates that lncRNAs have critical regulatory functions, such as ferroptosis [19], gemcitabine resistance [20], and glycolysis [21] in PAAD. In recent times, there has been a surge in interest regarding the investigation of tumor-derived extracellular vesicles and their payload, comprising diverse proteins and nucleic acids, including long noncoding RNAs (lncRNAs). These entities are increasingly recognized to play a pivotal role in the onset and progression of cancer [9, 22, 23, 24]. Our study systematically analyzed lncRNA expression datasets in PAAD samples from TCGA and GEO, identifying prognostic lncRNAs associated with extracellular vesicles.

Among these lncRNAs, LINC00996 and TRHDE-AS1 were selected for further scrutiny. LINC00996 has been identified as a potential lncRNA biomarker for early diagnosis across various cancer types, including colorectal cancer [25], bladder cancer [26], pulmonary adenocarcinoma and squamous cell carcinoma [27], ovarian cancer [28], and so on. Furthermore, Zhuan et al. [29] discovered that TRHDE-AS1 impedes the advancement of lung cancer through the miR-103/KLF4 axis. Meanwhile, Wei et al. [30] identified TRHDE-AS1 as an inhibitor of scar fibroblast proliferation via the miR-181a-5p/PTEN axis. However, investigations into cellular activities and the implications of these two signatures are currently lacking in PAAD. In our study, we found that LINC00996 is downregulated and functions as a beneficial factor in PAAD from different datasets and experimental evidence, which was consistent with previous research [31]. Similarly, TRHDE-AS1 showed results consistent with LINC00996 in our study, underscoring their crucial role in PAAD tumorigenesis and progression. Taken together, these research studies illustrate that the deletion of LINC00996 and TRHDE-AS1 represents crucial factors in the tumorigenesis and progression of PAAD.

Growing evidence suggests that extracellular vesicles act as a cargo transporter between cancer cells and immune cells [32, 33, 34]. In this study, patients with high expression of LINC00996 demonstrated increased immune cell infiltration across multiple datasets, encompassing B cells, CD8^+^ T cells, dendritic cells, and NK cells, alongside a more robust correlation with immune function. Simultaneously, our analysis revealed the significant involvement of natural killer cell-mediated cytotoxicity, T cell receptor, B cell receptor, and JAK-STAT3 signaling pathways in PAAD, as demonstrated by KEGG analysis. Furthermore, immune checkpoint blockade (ICB) was observed to augment the efficacy of antitumoral immune response through enhanced immune infiltration [35]. Notably, CD96, TIGIT, CTLA4, IDO1, and PDCD1 exhibited strong correlations with the high LINC00996 expression group, suggesting potential benefits from immunotherapy. On the contrary, elevated expression of TRHDE-AS1 displayed weak correlations with immune cell infiltration levels and immune function across various datasets, suggesting that TRHDE-AS1 may serve as an immunosuppression biomarker. Moreover, as demonstrated in our study, patients with high expression of LINC00996 and low expression of TRHDE-AS1 may derive greater benefits from anti-CTLA-4 and anti-PD-L1 therapy, consistent with prior findings. Overall, LINC00996 and TRHDE-AS1 hold the potential to accurately predict the prognosis of PAAD patients and guide personalized immunotherapy.

However, several limitations remain in the present study. First, we plan to undertake external validation of LINC00996 and TRHDE-AS1 expression in serum extracellular vesicles from PAAD patients. Second, our future endeavors will encompass conducting comprehensive phenomenological and molecular mechanism experiments to provide further insights into the observed findings.

## 5. Conclusion

In conclusion, our study focused on investigating exosome-associated lncRNA biomarkers to predict prognosis and guide personalized immunotherapy in PAAD patients. Targeting these lncRNAs holds promise for addressing systemic therapy failure and exploring novel pathways for immunotherapy. Consequently, the molecular mechanisms underlying the interactions between these lncRNAs and PAAD warrant further investigation.

## Data Availability

The PAAD RNA-seq dataset and Genotype-Tissue Expression (GTEx) project data utilized in this study are accessible through the UCSC Xena platform (http://xena.ucsc.edu/) and GEO datasets (https://www.ncbi.nlm.nih.gov/geo/). For additional inquiries, please contact the corresponding author.

## Supplementary Materials

Supplementary Materials Table S1: display of primer sequence used in RT-qPCR experiment.
