# Supplementary material for: Exploring Unique Extracellular Vesicles Associated Signatures: Prognostic Insights, Immune Microenvironment Dynamics, and Therapeutic Responses in Pancreatic Adenocarcinoma
Source: Mediators Inflamm. 2024 Aug 24;2024:2825971. doi: 10.1155/2024/2825971 (PMC11366062; doi:10.1155/2024/2825971)
Supplement: Supplementary Materials — Table S1: display of primer sequence used in RT-qPCR experiment. [file 2825971.f1.docx]

| qPCR primer | Sequence |
| --- | --- |
| human-LINC00996- forward | CTCTGCCACATCGTTCGGTTC |
| human-LINC00996- reverse | CTTCTTACGCTGCCAACTGCTAA |
| human-TRHDE-AS1- forward | GGGTGTAGAGAGGGAAGTTAGG |
| human-TRHDE-AS1- reverse | TTCTCTCCAGCTGCAGGGTGTA |
| human-GAPDH- forward | GTCTCCTCTGACTTCAACAGCG |
| human-GAPDH- reverse | ACCACCCTGTTGCTGTAGCCAA |
